# Expert consensus on the diagnosis and treatment of RET gene fusion non‐small cell lung cancer in China

**DOI:** 10.1111/1759-7714.15105

**Published:** 2023-09-18

**Authors:** Xingxiang Pu, Chunwei Xu, Qian Wang, Wenxian Wang, Fang Wu, Xiuyu Cai, Zhengbo Song, Jinpu Yu, Wenzhao Zhong, Zhijie Wang, Yongchang Zhang, Jingjing Liu, Shirong Zhang, Anwen Liu, Wen Li, Ping Zhan, Hongbing Liu, Tangfeng Lv, Liyun Miao, Lingfeng Min, Gen Lin, Long Huang, Jingping Yuan, Zhansheng Jiang, Chuangzhou Rao, Dongqing Lv, Zongyang Yu, Xiaoyan Li, Chuanhao Tang, Chengzhi Zhou, Junping Zhang, Hui Guo, Qian Chu, Rui Meng, Xuewen Liu, Jingxun Wu, Jin Zhou, Zhengfei Zhu, Weiwei Pan, Fei Pang, Jintao Huang, Kai Wang, Fan Wu, Tingting Shen, Shirui Zou, Bingwei Xu, Liping Wang, Youcai Zhu, Xinqing Lin, Jing Cai, Ling Xu, Jisheng Li, Xiaodong Jiao, Kainan Li, Huijing Feng, Lin Wang, Yingying Du, Wang Yao, Xuefei Shi, Xiaomin Niu, Dongmei Yuan, Yanwen Yao, Jing Kang, Jiatao Zhang, Chao Zhang, Jianfei Fu, Jianhui Huang, Yinbin Zhang, Pingli Sun, Hong Wang, Mingxiang Ye, Dong Wang, Zhaofeng Wang, Yue Hao, Zhen Wang, Bing Wan, Donglai Lv, Gang Lan, Shengjie Yang, Lin Shi, Yina Wang, Bihui Li, Zhang Zhang, Zhongwu Li, Yuan Li, Zhefeng Liu, Nong Yang, Huijuan Wang, Wenbin Huang, Zhuan Hong, Guansong Wang, Jiandong Wang, Meiyu Fang, Yong Fang, Xixu Zhu, Yi Shen, Yiping Zhang, Shenglin Ma, Yong Song, Yuanzhi Lu, Wenfeng Fang, Ziming Li, Lin Wu

**Affiliations:** ^1^ The Second Department of Thoracic Oncology, Hunan Cancer Hospital/The Affiliated Cancer Hospital of Xiangya School of Medicine, Central South University Central South University Changsha People's Republic of China; ^2^ Institute of Cancer and Basic Medicine (ICBM) Chinese Academy of Sciences Hangzhou People's Republic of China; ^3^ Department of Respiratory Medicine, Affiliated Jinling Hospital Medical School of Nanjing University Nanjing People's Republic of China; ^4^ Department of Respiratory Medicine Affiliated Hospital of Nanjing University of Chinese Medicine, Jiangsu Province Hospital of Chinese Medicine Nanjing People's Republic of China; ^5^ Department of Chemotherapy Chinese Academy of Sciences University Cancer Hospital (Zhejiang Cancer Hospital) Hangzhou People's Republic of China; ^6^ Department of Oncology, The Second Xiangya Hospital Central South University Changsha People's Republic of China; ^7^ Department of VIP Inpatient, Sun Yat‐Sen University Cancer Center, State Key Laboratory of Oncology in South China Collaborative Innovation Center for Cancer Medicine Guangzhou People's Republic of China; ^8^ Department of Cancer Molecular Diagnostics Core Tianjin Medical University Cancer Institute and Hospital Tianjin People's Republic of China; ^9^ Guangdong Lung Cancer Institute, Guangdong Provincial Laboratory of Translational Medicine in Lung Cancer, Guangdong Provincial People's Hospital Guangdong Academy of Medical Sciences, School of Medicine Guangzhou People's Republic of China; ^10^ State Key Laboratory of Molecular Oncology, Department of Medical Oncology, National Cancer Center/National Clinical Research Center for Cancer/Cancer Hospital Chinese Academy of Medical Sciences and Peking Union Medical College Beijing People's Republic of China; ^11^ Department of Medical Oncology, Lung Cancer and Gastrointestinal Unit, Hunan Cancer Hospital/The Affiliated Cancer Hospital of Xiangya School of Medicine Central South University Changsha People's Republic of China; ^12^ Department of Thoracic Cancer Jilin Cancer Hospital Jilin People's Republic of China; ^13^ Translational Medicine Research Center, Key Laboratory of Clinical Cancer Pharmacology and Toxicology Research of Zhejiang Province, Affiliated Hangzhou First People's Hospital, Cancer Center Zhejiang University School of Medicine Hangzhou People's Republic of China; ^14^ Department of Oncology Second Affiliated Hospital of Nanchang University Nanchang People's Republic of China; ^15^ Key Laboratory of Respiratory Disease of Zhejiang Province, Department of Respiratory and Critical Care Medicine, Second Affiliated Hospital of Zhejiang University School of Medicine, Cancer Center Zhejiang University Hangzhou People's Republic of China; ^16^ Department of Respiratory Medicine, Affiliated Drum Tower Hospital Medical School of Nanjing University Nanjing People's Republic of China; ^17^ Department of Respiratory Medicine Clinical Medical School of Yangzhou University, Subei People's Hospital of Jiangsu Province Yangzhou People's Republic of China; ^18^ Department of Medical Oncology Fujian Medical University Cancer Hospital & Fujian Cancer Hospital Fuzhou People's Republic of China; ^19^ Department of Pathology Renmin Hospital of Wuhan University Wuhan People's Republic of China; ^20^ Department of Integrative Oncology Tianjin Medical University Cancer Institute and Hospital Tianjin People's Republic of China; ^21^ Department of Radiotherapy and Chemotherapy, Hwamei Hospital University of Chinese Academy of Sciences Ningbo People's Republic of China; ^22^ Department of Pulmonary Medicine Taizhou Hospital of Wenzhou Medical University Taizhou People's Republic of China; ^23^ Department of Respiratory Medicine, the 900th Hospital of the Joint Logistics Team (the Former Fuzhou General Hospital) Fujian Medical University Fuzhou People's Republic of China; ^24^ Department of Oncology, Beijing Tiantan Hospital Capital Medical University Beijing People's Republic of China; ^25^ Department of Medical Oncology Peking University International Hospital Beijing People's Republic of China; ^26^ State Key Laboratory of Respiratory Disease, National Clinical Research Center for Respiratory Disease; Guangzhou Institute of Respiratory Health The First Affiliated Hospital of Guangzhou Medical University (The First Affiliated Hospital of Guangzhou Medical University) Guangzhou People's Republic of China; ^27^ Department of Thoracic Oncology, Shanxi Academy of Medical Sciences Shanxi Bethune Hospital Taiyuan People's Republic of China; ^28^ Department of Medical Oncology The First Affiliated Hospital of Xi'an Jiaotong University Xi'an People's Republic of China; ^29^ Department of Oncology, Tongji Hospital of Tongji Medical College Huazhong University of Science and Technology Wuhan People's Republic of China; ^30^ Cancer Center, Union Hospital, Tongji Medical College Huazhong University of Science and Technology Wuhan People's Republic of China; ^31^ Department of Oncology, the Third Xiangya Hospital Central South University Changsha People's Republic of China; ^32^ Department of Medical Oncology, the First Affiliated Hospital of Medicine Xiamen University Xiamen People's Republic of China; ^33^ Department of Medical Oncology, Sichuan Cancer Hospital & Institute, Sichuan Cancer Center, School of Medicine University of Electronic Science and Technology Chengdu People's Republic of China; ^34^ Department of Radiation Oncology Fudan University Shanghai Cancer Center Shanghai People's Republic of China; ^35^ Department of Cell Biology, College of Medicine Jiaxing University Jiaxing People's Republic of China; ^36^ Department of Medical Shanghai OrigiMed Co, Ltd Shanghai People's Republic of China; ^37^ Department of Medical Menarini Silicon Biosystems Spa Shanghai People's Republic of China; ^38^ Department of Medical Stone Pharmaceuticals (Suzhou) Co., Ltd. Shanghai People's Republic of China; ^39^ Department of Biotherapy, Cancer Institute First Affiliated Hospital of China Medical University Shenyang People's Republic of China; ^40^ Department of Oncology Baotou Cancer Hospital Baotou People's Republic of China; ^41^ Department of Thoracic Disease Diagnosis and Treatment Center, Zhejiang Rongjun Hospital The Third Affiliated Hospital of Jiaxing University Jiaxing People's Republic of China; ^42^ Department of Interventional Pulmonary Diseases Anhui Chest Hospital Hefei People's Republic of China; ^43^ Department of Medical Oncology, Qilu Hospital, Cheeloo College of Medicine Shandong University Jinnan People's Republic of China; ^44^ Department of Medical Oncology, Shanghai Changzheng Hospital Naval Medical University Shanghai People's Republic of China; ^45^ Department of Oncology, Shandong Provincial Third Hospital, Cheeloo College of Medicine Shandong University Jinan People's Republic of China; ^46^ Department of Pathology, Shanxi Academy of Medical Sciences Shanxi Bethune Hospital Taiyuan People's Republic of China; ^47^ Department of Oncology The First Affiliated Hospital of Anhui Medical University Hefei People's Republic of China; ^48^ Department of Interventional Oncology, The First Affiliated Hospital Sun Yat‐sen University Guangzhou People's Republic of China; ^49^ Department of Respiratory Medicine, Huzhou Hospital Zhejiang University School of Medicine Huzhou People's Republic of China; ^50^ Department of Shanghai Lung Cancer Center, Shanghai Chest Hospital Shanghai Jiao Tong University Shanghai People's Republic of China; ^51^ Department of Medical Oncology, Affiliated Jinhua Hospital Zhejiang University School of Medicine Jinhua People's Republic of China; ^52^ Department of Oncology Lishui Municipal Central Hospital Lishui People's Republic of China; ^53^ Department of Oncology, the Second Affiliated Hospital of Medical College Xi'an Jiaotong University Xi'an People's Republic of China; ^54^ Department of Pathology The Second Hospital of Jilin University Changchun People's Republic of China; ^55^ Senior Department of Oncology The 5th Medical Center of PLA General Hospital Beijing People's Republic of China; ^56^ Department of Radiation Oncology, Affiliated Jinling Hospital Medical School of Nanjing Nanjing People's Republic of China; ^57^ Department of Respiratory Medicine The Affiliated Jiangning Hospital of Nanjing Medical University Nanjing People's Republic of China; ^58^ Department of Clinical Oncology The 901 Hospital of Joint Logistics Support Force of People Liberation Army Hefei People's Republic of China; ^59^ Department of Thoracic Surgery Chuxiong Yi Autonomous Prefecture People's Hospital Chuxiong People's Republic of China; ^60^ Department of Respiratory Medicine, Zhongshan Hospital Fudan University Shanghai People's Republic of China; ^61^ Department of Oncology, The First Affiliated Hospital, College of Medicine Zhejiang University Hangzhou People's Republic of China; ^62^ Department of Oncology The Second Affiliated Hospital of Guilin Medical University Guilin People's Republic of China; ^63^ International Cooperative Laboratory of Traditional Chinese Medicine Modernization and Innovative Drug Discovery of Chinese Ministry of Education (MOE), Guangzhou City Key Laboratory of Precision Chemical Drug Development, School of Pharmacy Jinan University Guangzhou People's Republic of China; ^64^ Key Laboratory of Carcinogenesis and Translational Research (Ministry of Education/Beijing), Department of Pathology Peking University Cancer Hospital & Institute Beijing People's Republic of China; ^65^ Department of Pathology Fudan University Shanghai Cancer Center Shanghai People's Republic of China; ^66^ Department of Medical Oncology The Affiliated Cancer Hospital of Zhengzhou University, Henan Cancer Hospital Zhengzhou People's Republic of China; ^67^ Department of Pathology the First Affiliated Hospital of Henan University of Science and Technology Luoyang People's Republic of China; ^68^ Department of Medical Oncology, Jiangsu Cancer Hospital Nanjing Medical University Affiliated Cancer Hospital Nanjing People's Republic of China; ^69^ Institute of Respiratory Diseases, Xinqiao Hospital Third Military Medical University Chongqing People's Republic of China; ^70^ Department of Pathology, Affiliated Jinling Hospital Medical School of Nanjing University Nanjing People's Republic of China; ^71^ Department of Medical Oncology, Sir Run Run Shaw Hospital Zhejiang University Hangzhou People's Republic of China; ^72^ Department of Thoracic Surgery, Affiliated Jinling Hospital Medical School of Nanjing University Nanjing People's Republic of China; ^73^ Department of Oncology, Key Laboratory of Clinical Cancer Pharmacology and Toxicology Research of Zhejiang Province, Affiliated Hangzhou Cancer Hospital, Cancer Center Zhejiang University School of Medicine Hangzhou People's Republic of China; ^74^ Department of Clinical Pathology The First Affiliated Hospital of Jinan University Guangzhou People's Republic of China; ^75^ Department of Medical Oncology, Sun Yat‐sen University Cancer Center, State Key Laboratory of Oncology in South China Collaborative Innovation Center for Cancer Medicine Guangzhou People's Republic of China

**Keywords:** NSCLC, precision medicine, RET fusion, targeted therapy, tyrosine receptor kinase

## Abstract

The rearranged during transfection (RET) gene is one of the receptor tyrosine kinases and cell‐surface molecules responsible for transmitting signals that regulate cell growth and differentiation. In non‐small cell lung cancer (NSCLC), RET fusion is a rare driver gene alteration associated with a poor prognosis. Fortunately, two selective RET inhibitors (sRETi), namely pralsetinib and selpercatinib, have been approved for treating RET fusion NSCLC due to their remarkable efficacy and safety profiles. These inhibitors have shown the ability to overcome resistance to multikinase inhibitors (MKIs). Furthermore, ongoing clinical trials are investigating several second‐generation sRETis that are specifically designed to target solvent front mutations, which pose a challenge for first‐generation sRETis. The effective screening of patients is the first crucial step in the clinical application of RET‐targeted therapy. Currently, four methods are widely used for detecting gene rearrangements: next‐generation sequencing (NGS), reverse transcription‐polymerase chain reaction (RT‐PCR), fluorescence in situ hybridization (FISH), and immunohistochemistry (IHC). Each of these methods has its advantages and limitations. To streamline the clinical workflow and improve diagnostic and treatment strategies for RET fusion NSCLC, our expert group has reached a consensus. Our objective is to maximize the clinical benefit for patients and promote standardized approaches to RET fusion screening and therapy.

## Graphical abstract

Diagnosis and treatment process of RET gene fusion non‐small cell lung cancer in China.

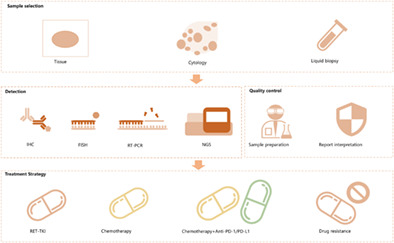

## INTRODUCTION

In the initial management of advanced non‐small cell lung cancer (NSCLC), patients with specific gene alterations such as *EGFR* mutation, ALK or ROS1 rearrangements are categorized as oncogene‐addicted tumors. These patients are typically treated with kinase inhibitors as the appropriate therapy. Conversely, patients without driver gene mutations or with other gene alterations are classified as nononcogene‐addicted tumors. For these patients, the recommended first‐line treatment options for them include chemotherapy and/or immunotherapy.^1^, ^2^

However, emerging evidence suggests that patients with oncogene alterations may achieve higher response rates when treated with selective inhibitors as their first‐line therapy, compared to chemotherapy. Additionally, targeted therapies have shown promise in extending survival and improving safety outcomes. Among the various oncogene drivers in NSCLC, rearranged during transfection (RET) rearrangement is a rare mutation found in only 1%–2% of NSCLC patients. Due to the structurally high similarity of RET and VEGFR2 ATP binding sites,^3^ several multikinase inhibitors (MKIs) have been tried for RET fusion NSCLC, such as cabozantinib, vandetanib, lenvatinib, and alectinib. However, these inhibitors have shown lower efficacy with a high safety risk, as evidenced by a median objective response rate (ORR) of approximately 30%, a median progression‐free survival (PFS) of 2.3 months, and a median overall survival (OS) of 6.8 months.^4^

Since 2016, BLU‐667 and LOXO‐292, two RET inhibitors, have presented promising preclinical data at international conferences, generating significant interest among clinical experts in RET‐altered tumors.^3^, ^5^ In 2020, both BLU‐667 and LOXO‐292 received FDA approval, marking the era of targeted therapy for RET fusion NSCLC.

Given the diversity of available testing platforms and the challenges associated within accumulating treatment experience due to the low incidence of RET fusion in NSCLC, it is crucial to standardize the screening of RET alterations and the application of RET inhibitors. In this expert consensus, we (1) recommend specific methods and platforms for RET gene testing, (2) propose treatment options based on these alterations, and (3) summarize the resistance mechanisms observed with first‐generation RET tyrosine kinase inhibitors (TKIs) and the progress in developing second‐generation RET TKIs.

## THE BIOLOGICAL BASIS OF THE *RET* GENE

### The gene structures and biological functions of the *RET* gene

**FIGURE 1:**
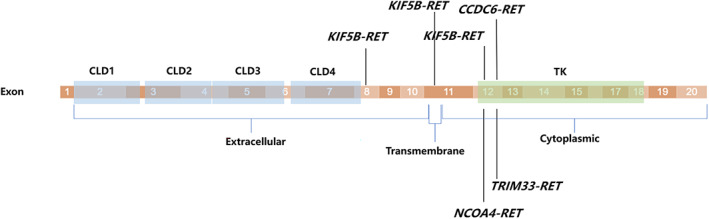
The structure of RET gene coding region. CRD, cysteine‐rich domain; GLD, 4 cadherin‐like domains; TK, tyrosine kinase domain.

The *RET* gene, located on the long arm of chromosome 10 (10q11.21), encodes a membrane tyrosine kinase receptor that plays a role in various cancers, including medullary thyroid carcinoma, papillary thyroid carcinoma, lung cancer, breast cancer, colorectal cancer, and so on. The RET protein consists of distinct regions, including an extracellular region encoded by exon 1–10 and part of exon 11, a transmembrane region encoded by part of exon 11, and a cytoplasmic region encoded by part of exon 11 and exon 12–19 or 12–20 (Figure 1). Notably, there is a cysteine‐rich domain (CRD, aa 515–634) that is crucial for receptor dimerization. While RET is primarily expressed in neural tissues such as the brain and autonomic nervous system, as well as in neuroendocrine cells and developing kidneys, it is also found in the lung, digestive tract, adult kidney, female and male reproductive organs, skin, and blood apparatus. In terms of physiological activation, the four RET ligands, namely GDNF, NRTN, PSPN, and ARTN, preferentially interact with coreceptors GFRA1, GFRA2, GFRA3, and GFRA4, respectively. These ligands form homodimers with a cystine knot at their center and require their respective coreceptors for RET activation.^6^

### *RET* gene alterations and their mechanism of carcinogenesis

To date, three primary mechanisms of aberrant RET activation have been identified in cancer: in‐frame *RET* gene fusions,^7^, ^8^ targeted mutations within the *RET* gene itself,^9^, ^10^, ^11^ and aberrant overexpression of the *RET* gene.^12^, ^13^ These abnormalities lead to the inappropriate activation of the RET tyrosine kinase even in the absence of its ligand. Consequently, the RAS‐MAPK and PI3K‐AKT signaling pathways are activated through the binding of adaptor proteins to phosphorylated sites on the RET protein.^14^, ^15^

The frequency of RET fusion in NSCLC is relatively low, ranging from 1% to 2%,^16^, ^17^, ^18^ with no significant difference observed between Asian and Western populations.^19^ Among the RET fusion cases, the most common partners are KIF5B and CCDC6, accounting for approximately 90% of cases.^20^ With the utilization of next‐generation sequencing (NGS) technology, over 50 distinct fusion partners have been identified, indicating a diverse and varied distribution in their occurrence. This demonstrates a clear long‐tail effect in the incidence distribution of RET fusion events.^21^

Activating point alterations represent another significant oncogenic mutation associated with inherited diseases such as multiple endocrine neoplasia 2A (MEN2A), multiple endocrine neoplasia 2B (MEN2B), and familial medullary thyroid carcinomas. These mutations are distributed across exons 7 to 16, predominantly occurring in the cysteine‐rich domain and tyrosine kinase domains. Noteworthy examples of these alterations include C609, C611, C618, C620 (exon 10), C630, D631, C634, T636, K666, D707 (exon 11), E505 (exon 7), C515, C531, G533, G548 (exon 8), E768, L790, Q781 (exon 13), V804 (exon 14), A883, S891, S904 (exon 15), M918, and R912 (exon 16).^6^

It is worth noting that RET fusion has been identified as one of the key mechanisms underlying acquired resistance to TKI treatments in cases involving other driver mutations.^4^ Following resistance to EGFR TKIs, the occurrence of acquired RET fusion was observed in approximately 1%–4% of patients, with CCDC6 being the predominant fusion partner.^22^, ^23^

## TYPES OF DETECTION METHOD AND THEIR LIMITATIONS

### Next‐generation sequencing (NGS)

In Asian NSCLC, especially lung adenocarcinoma,^19^ approximately 80% of patients carry driver gene mutations. Currently, more than 10 driver genes are known to be targetable or associated with resistance to targeted therapies. For patients with advanced NSCLC, tissue samples are typically obtained through biopsy, followed by histopathological examination using hematoxylin and eosin (HE) staining, immunohistochemical subtyping, and molecular testing. However, biopsies often yield limited amounts of tissue, necessitating the maximization of information extraction from scarce samples. The key advantage of NGS lies in its ability to simultaneously detect multiple genes and various types of mutations, including point mutations, fusions (rearrangements), and amplifications (copy number variations), even when sample quantities are limited.

With the increasing utilization and cost‐effectiveness of NGS technology, clinicians are increasingly acknowledging its value in identifying driver gene mutations in lung cancer patients, especially those experiencing drug resistance to targeted therapies. Nonetheless, the selection of an appropriate NGS panel necessitates careful consideration of clinical requirements, testing expenses, and turnaround time. For patients who have recently received a diagnosis, it is advisable to opt for a smaller panel comprising fewer than 100 genes. Conversely, for patients exhibiting treatment resistance, a larger panel may prove more advantageous.

### DNA‐based NGS

NGS offers notable advantages in terms of sensitivity and specificity, enabling the identification of gene alterations in both tissue and liquid samples. Hybrid capture and amplicon‐based technologies are two commonly employed methods in DNA‐based NGS. Currently, the targeted gene hybrid capture approach is primarily utilized in DNA‐based NGS for the detection of *RET* gene rearrangements, encompassing both known and unknown fusion partners. This is due to the absence of complex or repetitive sequences within the *RET* gene. In terms of RET fusion detection, NGS exhibits a sensitivity ranging from 87.2% to 100% and a specificity ranging from 98.1% to 100%.^24^, ^25^ However, it is crucial to acknowledge that the accuracy of gene fusion detection through NGS is influenced by critical factors such as the coverage of capture probes, sequencing depth, specimen quality, complexity of gene sequences, and bioinformatic analysis.^26^, ^27^

NGS detection utilizing amplicon technology offers the advantage of minimal DNA requirement and facilitates gene mutation testing. Nonetheless, it is important to acknowledge the limitations of this approach, as it can solely identify genes for which corresponding primer sequences have been designed. Consequently, amplicon‐based NGS may not be suitable for detecting unknown fusion partners or genes that lack identifiable primer sequences, thereby restricting its application.

Given the enhanced sensitivity, specificity, and clinical accessibility of DNA‐based NGS, it is commonly advised to employ this method for the identification of RET fusion in both newly diagnosed patients and those who have developed treatment resistance.

### RNA‐based NGS

RNA‐based NGS represents an optimal approach for the detection of gene fusions and exon skipping, as it focuses solely on retained exons at the RNA level, thereby simplifying the complexity of NGS testing. However, it is important to acknowledge the inherent instability and susceptibility to degradation of RNA, posing a significant challenge in maintaining high sample quality, particularly when working with formalin‐fixed paraffin‐embedded (FFPE) tissues. In a study, RNA‐based NGS sequencing was conducted on 55 samples with confirmed RET fusion positivity by DNA‐based NGS. Among these samples, 10 were excluded from the analysis due to inadequate sample quality.^28^ Among the remaining 44 samples with confirmed RET fusion positivity by DNA‐based NGS, 41 (93.2%) were successfully validated at the RNA level. Notably, in two cases where only a RET 5′‐end fusion was identified by DNA‐based NGS, RNA‐based NGS detected KIF5B‐RET (K24:R10) transcripts in one case, suggesting that DNA‐based NGS may overlook 3′‐end fusions.

According to available research findings, both DNA‐based NGS and RNA‐based NGS exhibit a high level of concordance in detecting RET fusion alterations. However, when considering factors such as economic cost and clinical accessibility, routine implementation of RNA‐based NGS for RET fusion gene testing is not advised. Nonetheless, in cases where DNA‐based NGS identifies a RET 5′‐end fusion, it is recommended to conduct further validation of the presence of the RET kinase domain using RNA‐based NGS.

### Reverse transcription‐polymerase chain reaction (RT‐PCR)

The rapid and straightforward detection of RET gene fusions is the primary advantage of RT‐PCR. However, its applicability is restricted to known fusion partners within the primer design range, thereby limiting its ability to identify unknown or novel fusion partners, which may result in false‐negative outcomes.^29^ RT‐PCR detects RET gene fusion at the RNA level, making the accuracy of the test results highly reliant on the RNA sample quality. Moreover, in terms of cost‐effectiveness, PCR testing is more advantageous compared to NGS. Therefore, in situations where NGS is not readily available, routine utilization of multiplex RT‐PCR for *RET* gene testing is recommended.

### Fluorescence in situ hybridization (FISH)

FISH is widely regarded as the gold standard technique for detecting gene rearrangements, regardless of the specific fusion partner involved. Its notable advantages include the ability to achieve single‐cell resolution and a rapid turnaround time. However, the cost of FISH testing remains relatively high, particularly when applied to single gene testing. Additionally, the interpretation of FISH results requires specialized technical expertise and is typically only accessible in larger medical centers. Furthermore, it is important to note that there is currently no universally standardized cutoff defining positivity in FISH analysis, as reported rates of RET split signals in positive cases range from 10% to 20% of cells.^30^, ^31^, ^32^

A comparative study assessing the efficacy of frequently employed assays in detecting RET fusions demonstrated that FISH exhibited a sensitivity of 100% for both KIF5B and CCDC6 but displayed a lower sensitivity of 66.7% for NCOA4.^33^ The diminished sensitivity observed for NCOA4 is attributed to the close proximity of the NCOA4 and RET genes.

In summary, although FISH demonstrates high sensitivity (100%) in detecting RET fusions, its specificity is suboptimal, ranging from 45% to 60%. As a result, FISH is not routinely recommended as a primary method for RET testing.

### Immunohistochemistry (IHC)

While IHC is a cost‐effective method suitable for large‐scale screening of ALK‐positive NSCLC,^34^ its effectiveness in detecting RET fusions is limited. A study demonstrated that RET IHC staining patterns showed no discernible differences between RET‐positive and RET‐negative specimens previously identified by RT‐PCR. The false‐negative (FN) and false‐positive (FP) rates for RET fusion detection by IHC were found to be 46% and 62%, respectively.^30^ Furthermore, three additional retrospective analyses^31^, ^35^, ^36^ yielded similar results, confirming the poor performance of IHC in detecting RET fusions. Consequently, IHC is not considered a reliable technique for the detection of RET fusions.

## TREATMENTS FOR RET FUSION NSCLC

Since 2020, the Food and Drug Administration (FDA) has granted approval for two selective RET tyrosine kinase inhibitors (sRETi) for the treatment of advanced RET NSCLC. These sRETi drugs are specifically designed to possess high potency and selectivity in targeting oncogenic RET alterations, including RET fusions such as KIF5B‐RET and CCDC6‐RET, as well as RET activating mutations like C634W, M918T, and V804L/M. The antitumor mechanism of sRETi involves competitive binding to the ATP receptor, thereby inhibiting the activity of the RET protein kinase.^37^, ^38^

### Pralsetinib

On September 4, 2020, pralsetinib (BLU‐667) received approval from the FDA for the treatment of adult patients with metastatic NSCLC positive for RET gene fusion.^39^ Following this, in March 2021, the National Medical Products Administration (NMPA) in China also granted approval for pralsetinib as the first selective RET tyrosine kinase inhibitor (sRETi) for the treatment of locally advanced or metastatic NSCLC with RET gene fusion in patients who had previously received platinum‐based chemotherapy. Subsequently, in June 2023, the NMPA approved pralsetinib as a first‐line treatment option for locally advanced or metastatic NSCLC with RET gene fusion.^40^

The ARROW study (NCT03037385) is a multicohort, open‐label, phase I/II clinical trial designed to assess the safety and effectiveness of pralsetinib in patients with advanced tumors harboring RET alterations. Updated data from the NSCLC cohorts, as published in the *Annals of Oncology*, revealed an overall response rate (ORR) of 79% (95% CI: 59–92) in treatment‐naïve patients and 59% (95% CI: 50–67) in patients who had previously received platinum‐based chemotherapy (data cutoff: November 6, 2020). The median duration of response (DoR) was not reached in treatment‐naïve patients and was 22.3 months in patients with prior platinum‐based chemotherapy. Among patients with measurable intracranial metastases, the intracranial response rate was 70% (7/10; 95% CI: 35–93), and the median DoR was 10.5 months (95% CI: 5.5–12.6).^41^

Further analysis of the relationship between RET fusion partners and treatment outcomes from the ARROW study confirmed the efficacy of pralsetinib in patients with RET fusion NSCLC, regardless of the specific fusion partner.^42^ The efficacy results of pralsetinib in Chinese patients were consistent with those reported in the global population.^43^

### Selpercatinib

Selpercatinib (LOXO‐292), another selective RET inhibitor (sRETi), received FDA and NMPA approval for the treatment of adult patients with metastatic NSCLC harboring RET gene fusions in May 2020 and October 2022, respectively.^44^, ^45^ Updated data was reported in the *Journal of Clinical Oncology* in 2022.^46^ The updated data set included a larger number of patients (*n* = 316) compared to the original reported population (*n* = 144). In treatment‐naïve patients, the objective response rate (ORR) was 84% (95% CI: 73–92). The median duration of response (DoR) was 20.2 months (95% CI: 13.0–could not be evaluated). The median progression‐free survival (PFS) was 22.0 months. In patients who had previously received platinum‐based chemotherapy, the ORR was 61% (95% CI: 55–67). The median DoR was 28.6 months (95% CI: 20.4–could not be evaluated). The median PFS was 24.9 months (*n* = 247, median follow‐up of 24.7 months). In the registrational analysis set, the median PFS was 19.3 months with longer follow‐up (*n* = 105, median follow‐up of 30.3 months). Among the 26 patients with measurable baseline CNS metastasis, the intracranial ORR was 85% (95% CI: 65–96), and the median DoR was 9.4 months (95% CI: 7.4–15.3).

### Options other than sRETi

#### Chemotherapy

Prior to the availability of selective RET TKIs (sRETi), platinum‐based chemotherapy was the standard treatment for RET fusion advanced NSCLC. However, there were no prospective chemotherapy trials specifically focused on RET‐fusion NSCLC due to the relatively low frequency of this genomic alteration.

A global, multicenter registry study was conducted to evaluate the efficacy of traditional chemotherapy in 84 patients who received platinum doublet as the first‐line treatment. The study showed that the overall response rate (ORR) was 51% (95% CI: 38.1–63.4), the median progression‐free survival (PFS) was 7.8 months (95% CI: 5.3–10.2), and the median overall survival (OS) was 24.8 months (95% CI: 13.6–32.3). Among the 66 patients who received a platinum agent in combination with pemetrexed, the ORR was 49% (95% CI: 35.4–62.9), the median PFS was 6.4 months (95% CI: 4.3–8.8), and the median OS was 23.6 months (95% CI: 13.6–32.3).^47^ Similar results were observed in a retrospective study conducted in China.^48^

These findings suggest that platinum‐based chemotherapy can lead to a certain level of response and survival benefit in patients with RET gene fusion NSCLC. However, the introduction of sRETi has revolutionized treatment options and has shown improved efficacy compared to traditional chemotherapy.

#### Is chemotherapy combined PD‐1/PD‐L1 inhibitors a better choice for RET‐fusion NSCLC?

The combination of platinum doublet and PD‐1/PD‐L1 inhibitors has become the standard treatment for patients without driver gene alterations.^49^, ^50^ However, previous phase III studies did not exclude patients with RET fusions from these trials. A post hoc subgroup analysis of patients treated with the CCP regimen (carboplatin, pemetrexed, and pembrolizumab) in the first‐line setting, including a small number of patients with RET fusions (*N* = 10), showed an ORR of 70% (7/10).^51^ In real‐world settings, a study of 12 patients with RET fusion NSCLC reported a median PFS of 5.4 months (1.4–14.2) and a median OS of 19 months (6.9‐not reached).^52^ These findings suggest that PD‐1/PD‐L1 inhibitors may not provide additional benefits beyond chemotherapy in patients with RET fusion NSCLC.

However, caution should be exercised when combining immune checkpoint inhibitor (ICI) therapy with targeted agents. Concurrent or sequential use of ICI therapy with targeted agents may increase the risk of severe immune‐related adverse events (irAEs).^53^ In the ongoing phase 1/2 LIBRETTO‐001 trial (NCT03157128), 22 out of 329 patients (7%) experienced hypersensitivity reactions attributed to selpercatinib, with a higher incidence observed in patients previously treated with ICIs (*n* = 17, 77%) compared to ICI‐naïve patients (*n* = 5, 23%).^54^ Therefore, our expert group has reached a consensus that ICI‐based therapy is not the optimal choice for RET fusion NSCLC, especially in patients who may consider selpercatinib treatment in the future.

### Treatment strategy for acquired RET fusion NSCLC

RET fusion can also be found in cases after EGFR/ALK TKI progression and appears to become more prevalent after the administration of third‐generation EGFR TKIs.^4^, ^22^ Piotrowska et al.^23^ demonstrated that the emergence of acquired RET fusion expression in NSCLC cell lines with *EGFR* mutations led to resistance against EGFR inhibitors, a challenge that could be overcome by combining EGFR and RET inhibition. Notably, two patients who harbored acquired RET fusion and were treated with pralsetinib and osimertinib exhibited PR, with ongoing treatment progress at the time the paper was authored. In a multicenter real‐world retrospective study involving 31 patients, two cohorts were formed based on whether pralsetinib‐based targeted therapy was initiated immediately upon detecting RET fusion subsequent to EGFR/ALK inhibitor progression. The results indicated a median time to treatment failure (mTTF) of 7.93 months for cohort 1 (*n* = 20) versus 4.24 months for cohort 2 (*n* = 11), with ORR of 35.0% and 18.2%, respectively. Generally, the combined treatment of pralsetinib and other TKIs was well tolerated, with adverse events aligning with the known profiles of the two drugs.^55^ Another prospective expanded access clinical trial aimed at patients with acquired RET fusion post‐osimertinib treatment involved the use of osimertinib and selpercatinib. This trial enrolled 14 patients, achieving an ORR of 50% and a median treatment duration of 7.9 months.^56^ These results underscore the potential of RET inhibitor‐based therapy as a promising strategy for overcoming acquired RET fusion in NSCLC patients.

### Drug resistance and second‐generation RET inhibitors

In the context of targeted treatment, the emergence of resistance to RET tyrosine kinase inhibitors (TKIs) is a common occurrence among patients. However, due to the relatively recent introduction of these medications to the market, the mechanisms underlying resistance to selective RET inhibitors have not been extensively studied. Nevertheless, several known variants have been identified that are associated with resistance to first‐generation RET inhibitors: (1) Solvent‐front mutations, such as RET G810S/C; (2) hinge region mutations, such as RET Y806C/C; (3) mutations in the “roof” region of the ATP binding site, such as RET L730; (4) bypass pathway mutations, such as MET/ERBB2/EGFR amplifications, *BRAF V600E*, *KRAS* mutations, *PIK3CA* mutation, ALK/ROS1 fusion; (5) small cell transformation of NSCLC and (6) other or unknown resistance mechanisms.^57^, ^58^, ^59^, ^60^, ^61^

Among these variants, solvent‐front mutations are the most common and recurring “on‐target” resistance mechanism, occurring in approximately 10% of patients who experience disease progression after sRETi treatment.^57^ To provide precise therapy for patients who develop resistance to sRETi, a second biopsy and genetic testing are recommended.

In response to drug resistance to first‐generation sRETi, four second‐generation RET TKIs have initiated clinical trials.^62^, ^63^, ^64^, ^65^ These second‐generation drugs, such as APS03118, TPX0046, LOXO‐260, and KL590586, were designed to target and overcome solvent‐front mutations. Preclinical data has demonstrated potent activity against solvent‐front and gatekeeper mutations, except for TPX0046, which does not show activity against gatekeeper mutations.^66^, ^67^, ^68^, ^69^ In the 2023 ASCO meeting, KL590586 presented the results of a phase I study, reporting an overall response rate (ORR) of 60% in the study population. Moreover, significant reductions in target lesions were observed in seven patients who had previously received first‐generation sRETi treatment.

## SUMMARY AND PROSPECTS

Although RET gene fusion is rare in NSCLC patients, its impact and burden should not be underestimated, particularly given the large population of advanced NSCLC patients in China. Our expert group thoroughly discussed the diagnosis and treatments of RET gene fusion NSCLC in China based on clinical practice **(**Table 1 **)**. The approval of sRETi by NMPA in China has significantly extended the lifespan of patients, emphasizing the importance of patient management.

**TABLE 1:** Expert consensus on the diagnosis and treatment of NSCLC patients with RET fusion in China.

	Consensus number	Key points	Recommendation level
Detection time point	Consensus 1	RET fusion testing is recommended for patients with stage III/IV NSCLC	Strongly recommended
	Consensus 2	RET fusion testing is recommended to be performed in conjunction with testing for other driver genes such as *EGFR*, *ALK*, *ROS1*, *BRAF*, *KRAS*, *HER2*, and so on	Strongly recommended
	Consensus 3	*EGFR*, *ALK*, and *ROS1* were tested first, when negative, RET gene fusion should be tested	Strongly recommended
	Consensus 4	RET gene fusion testing should be performed for patients who experience disease progression on targeted therapy with other driver gene mutations	Recommended
Detection method	Consensus 5	RET fusion testing is preferred to be performed using DNA‐based NGS	Strongly recommended
	Consensus 6	When NGS is unavailable, RET fusion testing can be performed using RT‐PCR	Recommended
	Consensus 7	When the tissue is very limited, RET fusion testing by FISH can be considered as an alternative	Recommended
	Consensus 8	When 5′‐end RET fusion is detected by DNA‐based NGS, RNA‐based NGS can be used to verify its activity	Recommended
	Consensus 9	RET protein expression tested by IHC	Not recommended
Detection strategy	Consensus 10	Tumor tissue or cytology samples are preferred for RET fusion testing. If it cannot be obtained or is insufficient, peripheral blood, pleural effusion, or cerebrospinal fluid can be considered for ctDNA testing in advanced NSCLC patients.	Strongly recommended
Detecting quality control	Consensus 11	Physicians should make every effort to obtain sufficient samples for both pathological and molecular diagnosis.	Strongly recommended
	Consensus 12	Laboratories should participate in annual quality control programs, such as the PQCC, CAP, CLIA, or other laboratory quality assessment initiatives.	Strongly recommended
Treatment strategy	Consensus 13	For RET gene fusion advanced NSCLC patients, treatment with sRETi drugs such as pralsetinib and selpercatinib is recommended.	Strongly recommended
	Consensus 14	For patients with acquired RET gene fusion, sequential treatment with sRETi following other TKIs could be a viable treatment option.	Recommended
	Consensus 15	When sRETi is not available or intolerable, platinum‐based chemotherapy ± bevacizumab is recommended as the first‐line treatment option.	Recommended
	Consensus 16	For RET gene fusion patients with resistance to sRETi, performing NGS testing to identify the mechanism of resistance is encouraged. This will help in determining whether second‐generation RET inhibitors or participation in related clinical trials are appropriate treatment options.	Recommended

Abbreviations: CAP, College of American Pathologists; CLIA, Clinical Laboratory Improvement Amendments; FISH, fluorescence in situ hybridization; IHC, immunohistochemistry; NSCLC, non‐small cell lung cancer; NGS, next‐generation sequencing; PQCC, Pathology Quality Control Center; RET, rearranged during transfection; RT‐PCR, reverse transcription polymerase chain reaction.

In the context of rare gene mutations, cost‐effective identification of target patients becomes crucial. Our expert consensus systematically compared the advantages and disadvantages of different testing platforms for RET gene fusion. DNA‐based NGS was identified as the first preferred method, followed by RT‐PCR, while FISH was considered as an alternative. IHC, however, was not recommended. For the treatment of patients with RET gene fusion NSCLC, sRETi should be the preferred option for the first‐line setting. If sRETi is unavailable, platinum‐double chemotherapy can be considered, but ICI monotherapy or combination with chemotherapy is not recommended for the first‐line setting in patients who may receive targeted therapy in the future.

Several critical areas still require further exploration. First, it is essential to establish a national platform for collecting Chinese patients' gene mutation data and refining the map of RET gene alterations in China. This will effectively translate the progress of molecular biology research into clinical practice benefits. Second, more high‐quality real‐world studies are needed for the two sRETi drugs. Efficacy and safety data from real‐world analysis will provide reliable experiences for the drugs' application. Last but not least, the resistance mechanism of sRETi, especially in Chinese patients, remains unclear and is highly relevant to subsequent treatment decisions. Therefore, exploratory studies on sRETi resistance and personalized treatment for patients are also necessary.

## AUTHOR CONTRIBUTIONS

Yuanzhi Lu, Wenfeng Fang, Ziming Li and Lin Wu participated in the design of the expert consensus. Xingxiang Pu, Chunwei Xu, Qian Wang, Wenxian Wang and Fang Wu conceived of the expert consensus, and participated in its design and other authors coordination and helped to draft the expert consensus. All authors read and approved the final manuscript.

## FUNDING INFORMATION

This work was supported by the Science and Technology Innovation Program of Hunan Province (grant number 2023SK4024), the Natural Science Foundation of China (grant number 82002456), China Postdoctoral Science Foundation (grant number 2022M723207), and the Medical Scientific Research Foundation of Zhejiang Province of China (grant number 2023KY666).

## CONFLICT OF INTEREST STATEMENT

There are no conflicts of interest to disclose.
